# Concentration-dependent responses of *C*. *reinhardtii* to silver ions: hormetic response in growth and reduction of motility

**DOI:** 10.1140/epje/s10189-025-00521-3

**Published:** 2025-09-15

**Authors:** Hemanta Pradhan, Arpan Poudel, Diksha Shrestha, Ariel Rogers, Michael Stewart, Amani Jereb, Jack Harper, Ming Li, Wen Zhang, Jingyi Chen, Yong Wang

**Affiliations:** 1 Department of Physics, University of Arkansas, Fayetteville, Arkansas 72701 USA; 2 Department of Electrical Engineering and Computer Science, University of Arkansas, Fayetteville, Arkansas 72701 USA; 3 Cell and Molecular Biology Program, University of Arkansas, Fayetteville, Arkansas 72701 USA; 4 Department of Chemistry and Biochemistry, University of Arkansas, Fayetteville, Arkansas 72701 USA; 5 Materials Science and Engineering Program, University of Arkansas, Fayetteville, Arkansas 72701 USA; 6 Department of Civil Engineering, University of Arkansas, Fayetteville, Arkansas 72701 USA

## Abstract

**Abstract:**

Elevated levels of silver in aquatic environments arising from widespread use of silver nitrate and silver nanoparticles in different sectors of industry and medicine pose significant biophysical challenges to aquatic microorganisms. Despite extensive toxicity studies of silver on bacteria and microbial communities, its influence on other aquatic microorganisms, such as microalgae, remains poorly understood. In this study, we investigated the biophysical response of *C. reinhardtii* microalgae to silver ion exposure in terms of their population growth dynamics, chlorophyll content, and swimming motility. We found that silver ions at different concentrations (from 0.29 to 1.18 $\upmu$M) elongated the lag phase of the microalgal growth. However, the growth of the microalgae was boosted by silver ions at low concentrations (e.g., 0.29 $\upmu$M), showing higher OD_750_ values at the stationary phase and higher maximum growth rates. This hormetic response exhibited by microalgae upon exposure to silver ions indicates a nonlinear coupling between ionic stress and cellular growth. Additionally, we quantified the chlorophyll content in the microalgae with different concentrations of silver ions using spectrophotometric analysis, which revealed that the microalgae cells contained twice as high concentrations of chlorophyll when exposed to silver ions at lower concentrations. More importantly, we monitored the motion of microalgae in the presence of silver ions, detected and tracked their motion using a deep learning algorithm, and determined the effects of silver ions on the swimming motility of individual *C. reinhardtii* microalgae. Our results showed reduced average swimming speed and increased directional change of microalgae upon silver ion exposure.

**Graphical abstract:**

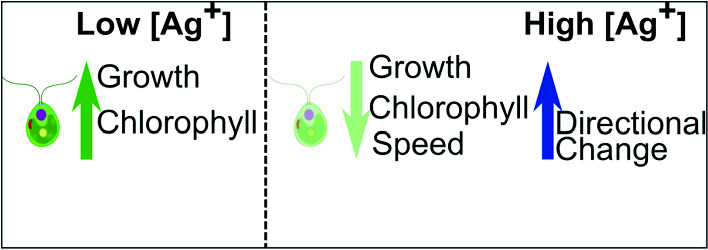

**Supplementary Information:**

The online version contains supplementary material available at 10.1140/epje/s10189-025-00521-3.

## Introduction

Silver (Ag) in various forms—ions and nanoparticles—has been extensively used in different fields: industry, agriculture, and medicine. For example, silver nitrate (AgNO_3_) and silver nanoparticles (AgNPs) have been the main agents for the treatment of wounds, burns, ulcers, warts, and chemical cauterization, due to their antimicrobial properties, i.e., toxic effects to microbes [1, 2]. They have been widely used in various industrial sectors, including electroplating, mirror-making, production of dyes, inks, textile production, and water treatment [3–6]. Additionally, AgNO_3_ and AgNPs have been used in agriculture for plant growth control, flowering development, and agricultural pest control [3, 5–7]. Due to the wide uses of silver in different forms, the release of silver ions (Ag^+^) into the environment (e.g., freshwater bodies)—through industrial discharges, agricultural runoffs, and wastewater releases—has been an emerging issue, because of the toxic effects of Ag^+^ ions to microorganisms [8–11]. To assess the impact of Ag^+^ ions on aquatic ecosystems, it is important to understand how they affect the growth, viability, and behaviors of aquatic microorganisms.

Efforts have been made to evaluate the toxic effects of Ag^+^ ions and AgNPs on freshwater and marine microorganisms, such as microalgae, which play essential roles in the aquatic ecosystems. It was reported that both Ag^+^ ions and AgNPs suppressed the growth of various microalgae, including *Chlamydomas reinhardtii* (*C. reinhardtii*), *Chlorella vulgaris*, *Chara vulgaris,* and *Pithophora oedogonia* [8, 10–12]. For *C. reinhardtii*, a commonly used model system in biophysics and photosynthesis research, the half maximal effective concentration (EC_50_) of Ag^+^ ions was reported to be $\sim$0.2 $\upmu$M, while that of AgNPs was 1 $\upmu$M [10]. It was also shown that cell viability, photosynthetic yield, and chlorophyll content were significantly reduced when the microalgae were exposed to Ag^+^ ions and AgNPs [8, 10–12]. Despite most studies reporting adverse effects of Ag^+^ ions and AgNPs, their effects may be more complicated and controversial. For example, it was reported Ag at low concentrations showed beneficial effects on the growth of sugarcane, increasing shoot length and number [13]. An immediate question is whether such hormetic effects of Ag exist on microalgae.

Beyond cell growth and viability, motility is a fundamental biophysical trait of microalgae (e.g., *C. reinhardtii*) for them to find nutrients and light, and to escape from hazards [14–18]. The motility of such microalgae is governed by coordinated flagellar motion and influenced by physical forces and environmental signals. However, studies on the impact of Ag^+^ ions and AgNPs on the motility of microalgae are limited. Several studies reported that metal oxide nanoparticles (e.g., TiO_2_, ZnO, and Fe_2_O_3_) and heavy metal ions (e.g., copper and cadmium) impair the motility of marine microalgae (e.g., *Platymonas subcordiformis* and *Platymonas helgolandica var. tsingtaoensis*) by reducing their curvilinear velocity, average path velocity (VAP), swimming linearity, and mean percentage of motile cells (MOT%) [15, 19]. However, the effects of Ag on microalgal motility are not clear. Additionally, most studies in the literature focused on the motility of ensemble groups of microalgae, while analysis of the effects of heavy metals on the motility of individual microalgae in response to ionic perturbation was much rarer.

In this study, we investigated the effects of Ag^+^ ions on the growth, chlorophyll content, and individual motility of *C. reinhardtii* microalgae. With a wide range of concentrations of Ag^+^ ions, we observed a hormetic response in growth kinetics. We also quantified the chlorophyll content in the microalgae exposed to Ag^+^ ions of different concentrations. Additionally, we observed the swimming of microalgae under a microscope, tracked the motion of individual microalgal cells using deep learning algorithms, quantified their speeds and directional changes, and evaluated the effects of Ag^+^ ions on the motility of microalgae. The findings in this work enhanced our understanding of microalgae responses to silver ions, providing insights on interactions of freshwater microorganisms with heavy metal ions.

## Materials and methods

### *C*. *reinhardtii* strain and culture

A wild type *C. reinhardtii* strain (CC-125 wild type mt+ [137c]) was purchased from the Chlamydomonas Resource Center (University of Minnesota, MN, USA), and used in this study. The microalgae were grown in tris-acetate-phosphate (TAP) medium (50 mL) in Erlenmeyer flasks on a 14 hr–10 hr day–night cycle (day time = 6 am–8 pm, night time = 8 pm–6 am) in a shaking incubator with 180 rpm at a temperature of 24 $^\circ$C [20]. The light intensity during the daytime was 118 $\upmu$mol/m$^2$s. During the night time, the light was turned off, and the intensity was reduced to zero.

### Growth curve assays of microalgae in the presence of Ag^+^ ions

We measured the growth curves of microalgae [9, 11] in the presence of Ag^+^ ions at concentrations of 0, 0.29, 0.59, 0.88, and 1.18 $\upmu$M (corresponding to concentrations of AgNO_3_ at 0, 0.05, 0.10, 0.15, to 0.20 $\upmu$g/mL). Briefly, the stock microalgae (maintained in TAP medium) were diluted and regrown in fresh TAP medium, under the described culturing conditions, to reach an optical density at 750 nm (OD_750_) of 0.7$-$0.8, followed by dilution in fresh TAP medium (6 mL) with Ag^+^ ions at different concentrations again to reach OD_750_ = 0.01. Ag^+^ ions were introduced by adding fresh AgNO_3_ solutions (58.9 mM or 10 $\upmu$g/mL) to the TAP medium to the desired final concentrations. The diluted samples were maintained under the described culturing conditions for 188 h, while the OD_750_values of these samples were monitored every 12 h using a UV–vis spectrophotometer (Implen NanoPhotometer C40, Implen GmbH, Munich, Germany). Six replicates were measured for each sample (i.e., at each concentration of Ag^+^ ions).

### Chlorophyll content quantification after exposure to Ag^+^ ions

After the microalgae cells were exposed to Ag^+^ ions at different concentrations (0, 0.29, 0.59, 0.88, and 1.18 $\upmu$M) for 188 h, the total chlorophyll contents were quantified. Briefly, 1 mL of each sample was centrifuged for 10 min at 13,000 rpm ($\sim 16,000$ g), followed by discarding the supernatant. The pellets were then mixed with 1 mL of a mixture solution of acetone (80% v/v) and methanol (20% v/v), and vortexed for 30 sec, followed by centrifugation at 13000 rpm for 7 min. The supernatants were collected for quantifying the concentration of chlorophyll content by measuring the absorbance at 663 nm, 645 nm, and 750 nm [21],

1 $$\begin{aligned}&\text {Chl a }({\upmu }\text {g/mL}) = \frac{12.25 E_{663} - 2.55 E_{645}}{\text {Sample volume (mL)}} \end{aligned}$$

2 $$\begin{aligned}&\text {Chl b }({\upmu }\text {g/mL}) = \frac{20.31 E_{645} - 4.91 E_{663}}{\text {Sample volume (mL)}} \end{aligned}$$

3 $$\begin{aligned}&\text {Chl a+b }({\upmu }\text {g/mL}) = \frac{17.76 E_{645} + 7.34 E_{663}}{\text {Sample volume (mL)}} \end{aligned}$$

where $E_{663}$ and $E_{645}$ correspond to the measured absorbances at 663 nm and 645 nm, minus that at 750 nm, respectively. Then, the normalized chlorophyll content were estimated by dividing the values by the cell numbers using (1 OD_750_ $\approx$ 10^7^ cells/mL) [22].

### Single-cell swimming motility assay of microalgae in the presence of Ag^+^ ions

**Fig. 1:**
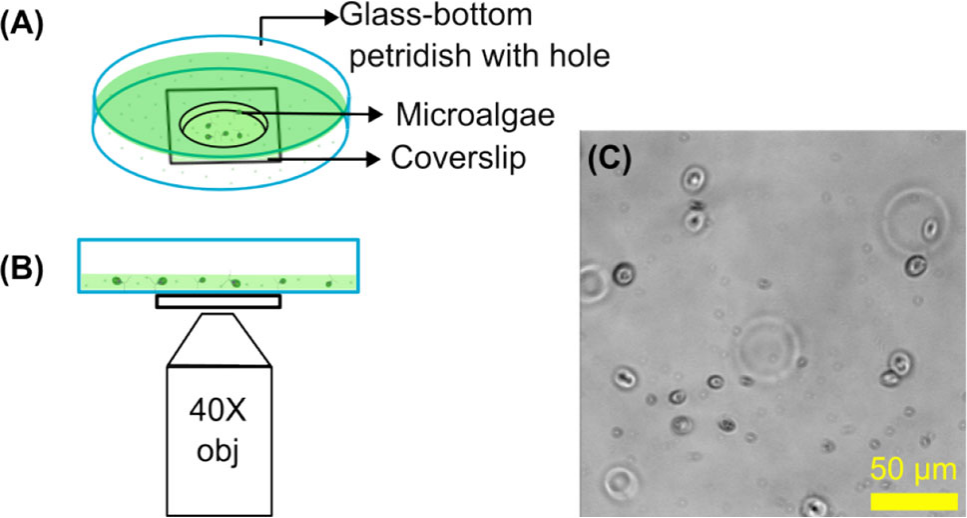
Illustration for sample preparation and imaging. **A** Schematic diagram of the imaging chamber made from a petri dish with a hole and glued to a glass coverslip. **B** Sketch for imaging microalgae under a microscope with a 40$\times$ objective. **C** An example of raw image of the microalgae under the microscope

We measured the single-cell swimming motility of microalgae under a microscope in the presence of Ag^+^ ions at different concentrations (0, 1.77, and 2.35 $\upmu$M), corresponding to concentrations of AgNO_3_ at 0, 0.3, and 0.4 $\upmu$g/mL. Briefly, fresh TAP agar plates were first streaked using colonies from older TAP agar plates of the *C. reinhardtii* microalgae. Colonies from the agar plates were picked and inoculated into fresh liquid TAP media (50 mL) for growth under the described culturing conditions. As the OD_750_ of the fresh culture reached $\sim 0.6$ (around 9 am), 10 mL of the samples were collected, and settled down at room temperature for 15 min, followed by harvesting 2 mL of the samples from the top layers. We note that this step is essential to remove non-motile microalgae and to acquire consistent motility results. Then, 2 mL of the harvested microalgae samples were added to glass-bottom petri dishes (Catalog #: PDH00001-200, Cell E&G LLC, USA; Fig. 1A), which were cleaned by NaOH solution (1 M), ethanol (100%), and pure water, and coated with bovine serum albumin (BSA, 1% for 20 min). The petri dishes were then mounted on an inverted microscope (Olympus IX-73, Olympus Corporation, USA) equipped with a 40X phase contrast objective (Olympus UPLFLN40XPH-2, NA=0.75; Fig. 1B) and an EMCCD camera (Andor Technology, USA). After taking a video at time 0, Ag^+^ ions (in the form of 80 $\upmu$L fresh AgNO_3_ solution) were added into the samples by inserting the pipette tips into the sample ($\sim$ 1 mm above the top surface of the coverslips of the petri dishes) right above the center of the objective and gently pushing out the solution without pipetting up and down. Videos of the microalgae were acquired under the microscope at 0, 15, 30, 45, and 60 min (Fig. 1C). The microscope and video acquisition were controlled by $\upmu$Manager [23, 24]. The effective pixel size of the acquired videos was 0.4 $\upmu$m, while the field of view was 512 by 512 pixels. The exposure time was set to 15 ms, while the actual interval between frames was 39.60 ms. Each video was taken for 5,000 frames. Five replicates were carried out for each sample (i.e., for each concentration of Ag^+^ ions and for each treatment duration).

**Fig. 2:**
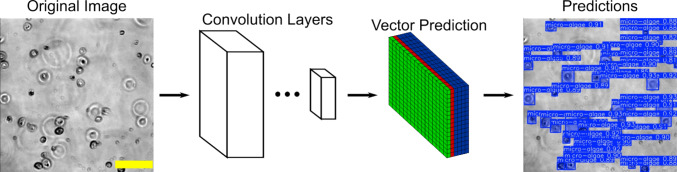
Illustration of automated detection of microalgae using the YOLO model

**Fig. 3:**
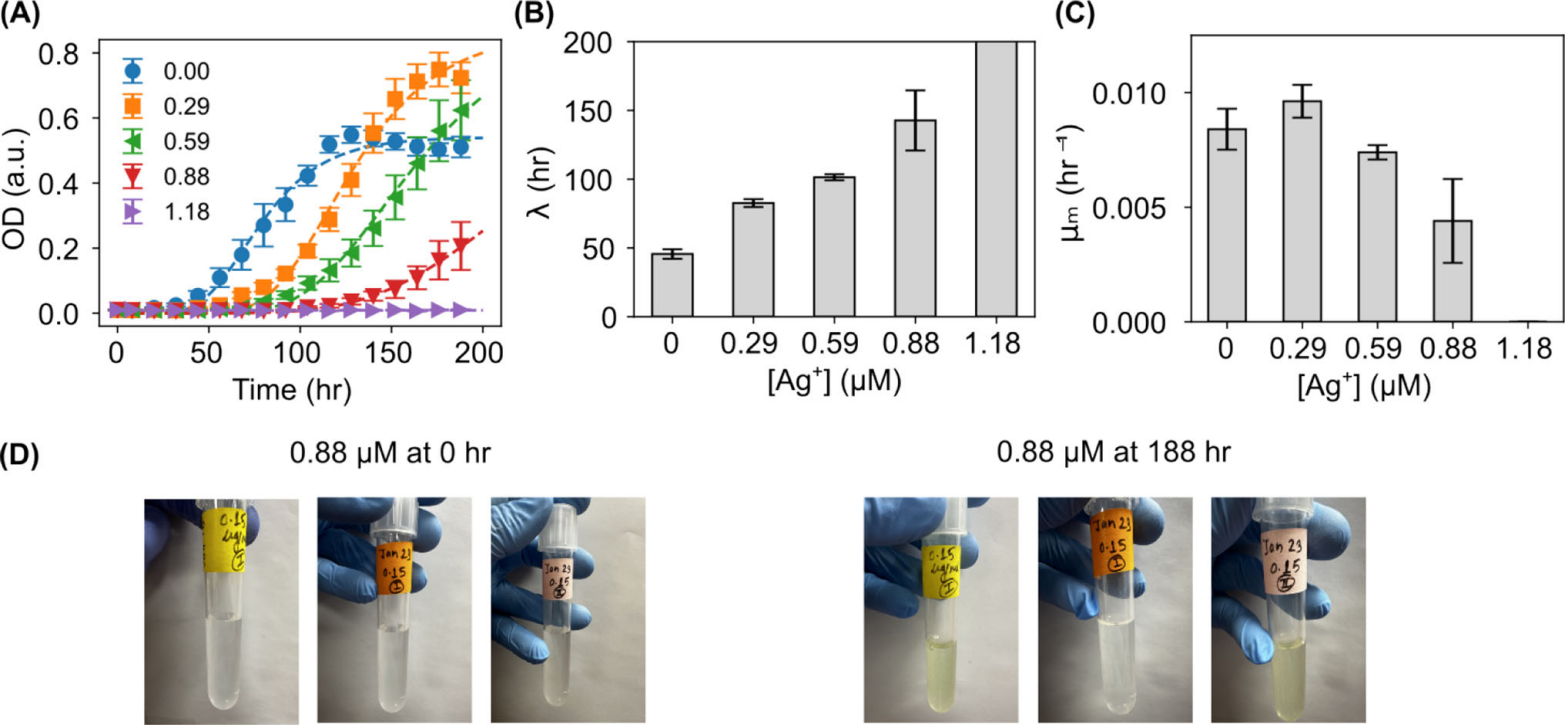
Growth curve assay of *C. reinhardtii* microalgae upon exposure to Ag^+^ ions at different concentrations. **A** Growth curves of the microalgae with Ag^+^ ions at different concentrations (0$-$1.18 $\upmu$M). Dashed lines are fitting curves using the Gompertz model. **B** Dependence of the fitted lag time ($\lambda$) on Ag^+^ concentration. **C** Dependence of the fitted maximum growth rate ($\mu _m$) on Ag^+^ concentration. **D** Visual demonstration of the high heterogeneity of microalgal growth at 0.88 $\upmu$M Ag^+^ ions at the end (188 hr) of the growth curve assay

### Deep learning based, automated detection and tracking of microalgae

We adopted YOLO (You Only Look Once), an object detection system based on convolutional neural networks [25], and trained our own model for automated detection of microalgae cells in the acquired videos. To train the model, 671 images were randomly selected from the acquired videos for manual annotation of the microalgal cells using the VGG image annotator (VIA) [26]. A total of 21,319 cells were manually annotated. The annotated images were split into training (87%), validation (9%), and testing (3%) datasets, converted to the YOLOv8 format [27] using Roboflow [28], and used for training our own microalgae detection model on an NVIDIA V100 GPU. A stochastic gradient descent (SGD) optimizer with an initial learning rate of 0.01 and a momentum of 0.937 was used for the model training, while the number of epochs was set to 2000 and the batch size was set to 128. The trained model was first verified by the validation and testing datasets and then applied to all the frames of acquired videos, which detected the microalgal cells and predicted their x and y positions, areas, and frame numbers (Fig. 2). The (x, y) positions and frame numbers of the microalgae were lastly linked into trajectories using *Trackpy* [29], with a maximum displacement of 15 pixels (6 $\upmu$m) and a memory of 3 frames.

### Analysis of the motility of microalgae in the presence of Ag^+^ ions

To evaluate the motility of the microalgae, we calculated the instantaneous velocities and directional changes from their trajectories, $\vec {r}(t)$. Briefly, the instantaneous velocities and their magnitudes (i.e., the instantaneous speeds) were evaluated by $\vec {v}(t) = \frac{\vec {r}(t+\Delta t) - \vec {r}(t)}{\Delta t}$ and $v(t) = \left| \vec {v}(t) \right|$, respectively, where $\Delta t = 0.0396$ s is the time interval between consecutive frames [30]. Directional changes were calculated by $\delta \theta = \cos ^{-1}\left( \frac{\vec {v}(t+\Delta t) \cdot \vec {v}(t)}{v(t+\Delta t) v(t)} \right)$ [31].

## Results & discussions

### Ag^+^ ions caused elongated lag time of microalgae

We measured the growth curves of the *C. reinhardtii* microalgae in the presence of Ag^+^ ions at different concentrations (0$-$1.18 $\upmu$M) for 188 hr. Started with an OD_750_ = 0.01, the control (untreated, 0 $\upmu$M) showed the classical sigmoidal growth curve (Fig. 3A). Fitting the growth curve with the Gompertz model, $y = A \exp \left\{ -\exp \left[ \frac{\mu _m e}{A} (\lambda - t) + 1 \right] \right\}$ [32, 33], gave a lag time of 45.6±3.5 hr (Fig. 3A and B). Compared to the control (0 $\upmu$M), the growth curves of the microalgae exposed to Ag^+^ ions shifted to the right, resulting longer lag times (82.6±2.8 hr, 101.3±2.2 hr, and 142.7±21.8 hr for the microalgae with Ag^+^ ions at 0.29, 0.59, and 0.88 $\upmu$M, respectively), as shown in Fig. 3A and B. At the concentration of 1.18 $\upmu$M of Ag^+^ ions, the microalgae did not grow in 188 hr. Our observation was consistent with previous studies in the literature [34, 35]. It was reported that Ag^+^ ions could damage various cellular components (e.g., membrane, DNA, and proteins), generate reactive oxygen species (ROS), and disrupt cellular functions (e.g., ATP synthesis) [9, 10, 35, 36]. It is thus expected that the microalgae exposed to Ag^+^ ions took time and effort to repair such damages and to adapt to the Ag^+^-containing environment, leading to elongated lag time in the growth curves.

Interestingly, a closer examination showed larger error bars in the growth curves of the microalgae exposed to Ag^+^ ions at 0.59 and 0.88 $\upmu$M (Fig. 3A). Similarly, a significantly larger error bar of the fitted lag time was present for the microalgae treated with 0.88 $\upmu$M Ag^+^ ions (Fig. 3B). This observation indicates that high heterogeneity exists in the response of microalgae to 0.88 $\upmu$M Ag^+^ ions, which was confirmed by visual examination of the color of the microalgae with Ag^+^ ions at this concentration: the microalgae grew and appeared green sometimes while not always under the same condition (Fig. 3D). A similar large heterogeneity was reported previously for the response of bacteria to silver ions [37].

### Ag^+^ ions at low concentrations led to hormetic response of microalgae growth

Another interesting observation is the hormetic response of the microalgae growth to Ag^+^ ions at low concentrations (0.29 and 0.59 $\upmu$M). As shown in Fig. 3A and Supplementary Figure 1, the asymptote (maximal value, or plateau value, of the sigmoidal growth curves) reached by the microalgae in the presence of 0.29 $\upmu$M Ag^+^ ions (0.87) was higher than that of the control (0.54). Although the microalgae with 0.59 $\upmu$M Ag^+^ ions did not reach the stationary phase in 188 hr, the OD_750_ value has already been higher than the asymptote of the control. The fitted maximum growth rate ($\upmu$_m_) of the microalgae with 0.29 $\upmu$M Ag^+^ ions was similar to that of the control (0.0096±0.0007 hr^-1^ *vs.* 0.0084±0.0009 hr^-1^; Fig. 3C). Although it is well known that many essential minerals (e.g., Phosphorus, Copper, Zinc) can have hormetic effects [38–40], reports on the hormetic effects of non-essential minerals are much rarer. Nonetheless, hormesis was observed in sugarcane multiplication *in vitro* responding to AgNPs [13, 41]. It was revealed that the presence of AgNPs enhanced the uptaking of macronutrients and micronutrients, which are associated with the biosynthesis and chlorophyll and presumably contribute to the enhanced growth. It was also suggested that responses to the ROS caused by AgNPs raised antioxidant capacity in sugarcane [13], while microalgae could respond to membrane damages caused by AgNPs by raised secretion of extracellular polymeric substances (EPS). Both these responses may better prepare the cells for and protect them from additional damages [42, 43]. As Ag$^+$ ions and AgNPs share some common cellular damaging mechanisms (e.g., ROS generation, membrane disruption) [44, 45], it is therefore not impossible that Ag^+^ ions are able to stimulate the growth of the microalgae at low concentrations.

### Ag^+^ ions at sublethal concentrations increased chlorophyll content in microalgae

**Fig. 4:**
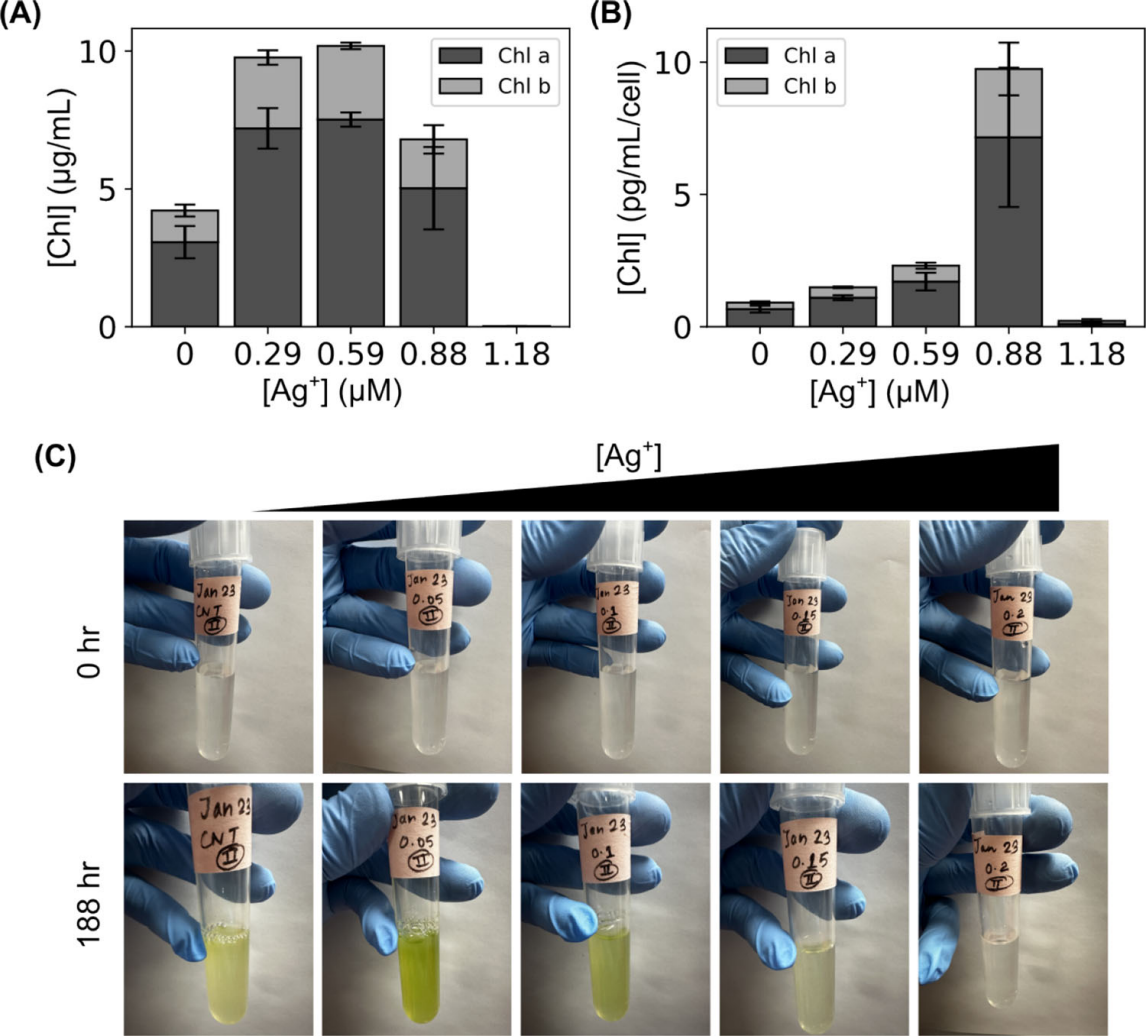
Chlorophyll content quantification in the *C. reinhardtii* microalgae upon exposure to Ag^+^ ions at different concentrations. **A** Dependence of the total chlorophyll content in the samples on Ag^+^ concentration. **B** Dependence of the chlorophyll content per cell (i.e., normalized by cell number) on Ag^+^ concentration. **C** Visual demonstration of the higher chlorophyll content at low concentration of Ag^+^ ions after 188 hr

It is controversial how silver affects the chlorophyll content in microalgae and plants. Some studies showed that AgNPs decreased the total chlorophyll content (chl a + chl b) in microalgae, *C. vulgaris* [12, 46], while other studies reported that AgNPs at similar or higher concentrations increased the total chlorophyll content [35, 47]. To better understand the observed hormetic effects of Ag^+^ ions on microalgal growth, we measured the chlorophyll content (chl a and chl b) after exposing the microalgae for 188 hr. As shown in Fig. 4A, the total chlorophyll content (chl a + chl b) in microalgae exposed to sublethal concentrations of Ag^+^ ions (0.29, 0.59, and 0.88 $\upmu$M) was much higher than the control. Particularly, the samples with 0.29 and 0.59 $\upmu$M Ag^+^ ions showed more than twice higher concentrations of total chlorophyll content (9.8 ± 1.0 and 10.2 ± 0.3 $\upmu$g/mL, respectively) than that of the control without Ag^+^ ions (4.2 ± 0.8 $\upmu$g/mL). The higher chlorophyll content in these samples could be confirmed visually, as they appeared greener (Fig. 4C). Although the growth of the microalgae was significantly suppressed by Ag^+^ ions at 0.88 $\upmu$M (Fig. 3A), the total chlorophyll content in this sample (6.8 ± 2.0 $\upmu$g/mL) was 62% higher than that of the control. In addition, we evaluated the chlorophyll content per microalgal cell (i.e., normalized by cell number) by dividing the measured concentration of chlorophyll content by the cell number inferred from the OD_750_ (1 OD_750_ $\approx$ 10^7^ cells/mL) [22]. We confirmed that the Ag^+^-exposed microalgae (except the one with 1.18 $\upmu$M Ag^+^ ions where no growth was observed) showed much higher chlorophyll content per cell (1.5 ± 0.1 pg/mL per cell, 2.3 ± 0.4 pg/mL per cell, and 9.7 ± 3.6 pg/mL per cell for Ag^+^-concentrations of 0.29 $\upmu$M, 0.59 $\upmu$M, and 0.88 $\upmu$M, respectively, compared to the control of 0.9 ± 0.2 pg/mL per cell), as shown in Fig. 4B. Therefore, our result supports that silver increased chlorophyll content [35], presumably better preparing the microalgae to adapt to the silver-containing environment and accounting for the stimulated growth with Ag^+^ ions at low concentrations [48, 49].

**Fig. 5:**
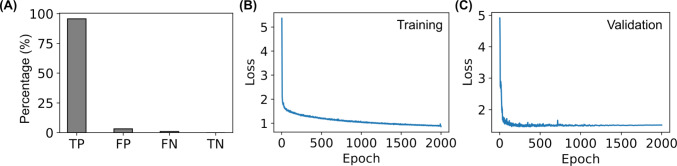
Accuracy and metrics of the custom-trained YOLO model for automated detection of *C. reinhardtii* microalgae. **A** The percentage of detected true positive (TP), false positive (FP), false negative (FN), and true negative (TN) using the trained YOLO model. **B**, **C** Loss curves for the training and validation datasets, respectively

**Fig. 6:**
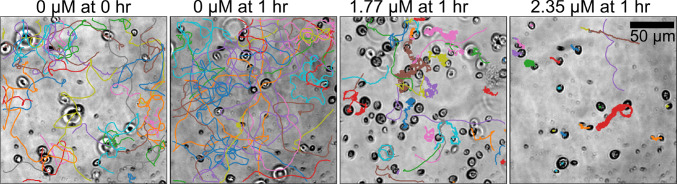
Examples of microalgal trajectories (colored curves) in the absence (0 $\upmu$M) and presence (1.77 $\upmu$M and 2.35 $\upmu$M) of Ag^+^ ions for 1 hr. Each panel contains 40 randomly chosen trajectories

### Automated detection and tracking of microalgae were successfully achieved by custom-trained YOLO model

Deep learning algorithms based on convolutional neural networks were exploited to automatically detect and track microalgae in our microscopic studies of the individual motility of the microalgae. YOLO (You Only Look Once) [50], one of the most popular object detection frameworks, was selected in the current study. It has been used for detection of microalgae previously [51–55]. A custom YOLO model was trained with our own data for detecting microalgae, as described in the Section of “Materials and Methods.” At the end of the training of 2000 epochs (with a batch size of 128), all the loss curves for the training and validation datasets converged (Fig. 5B and C), indicating the model reached a stable stage at the end of the training [56, 57]. Additionally, the custom-trained model applied to the testing dataset gave a high true positive rate of 96% and very low rates ($<1$%) for false positives and false negatives. The trained model did not detect any true negatives (0%). These verifications showed that our custom-trained model is accurate and sensitive for detecting *C. reinhardtii* microalgae cells.

**Fig. 7:**
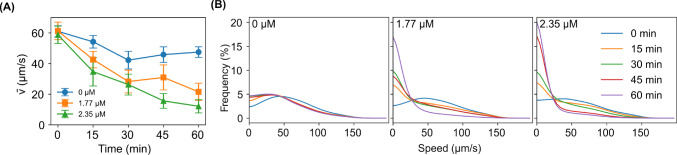
Comparison of microalgal speeds upon exposure to Ag^+^ ions at different concentrations for different durations. **A** Dependence of the average speed ($\bar{v}$) of the microalgae on the exposure time in the absence (0 $\upmu$M) and presence (1.77 and 2.35 $\upmu$M) of Ag^+^ ions. **B** Distributions of instantaneous speeds of microalgae at different concentrations of Ag^+^ ions for different durations

We applied the custom-trained YOLO model to detect the microalgae in every frame of all the acquired videos after exposure to Ag^+^ ions at different concentrations (0 $\upmu$M – control, 1.77 $\upmu$M, and 2.35 $\upmu$M) for durations from 0 min to 60 min at 15 min intervals. The concentrations of Ag^+^ ions used in the motility assay were slightly higher than the maximum concentration used in the growth curve assay (1.18 $\upmu$M), because the exposure time (60 min) was much shorter than that for the growth curve assay (188 hr) and Ag^+^ ions below 1.18 $\upmu$M did not visually affect the motility of the microalgae in 60 min. The x/y positions of the detected microalgae by our YOLO model were linked into trajectories using *Trackpy* [29] with a maximum displacement of 15 pixels and a memory of 3 frames. Random examples of the trajectories are shown in Fig. 6. Each colored curve represents a trajectory of a single microalga, while single frames of the acquired videos are shown as the backgrounds. Compared to the control, the microalgae exposed to Ag^+^ ions showed a higher frequency of confined motions and large directional changes, indicating that Ag^+^ ions inhibited the movement of the microalgae.

### Ag^+^ ions reduced the speed of microalgae

To further confirm that Ag^+^ ions suppressed the motion of the microalgae, we calculated the instantaneous velocities ($\vec {v}$) and speeds ($v=|\vec {v}|$) of the microalgae directly from the trajectories. The dependence of the average speed on the exposure time is shown in Fig. 7A. The average speed decreased significantly in the presence of Ag^+^ ions. For example, Ag^+^ ions at 2.35 $\upmu$M reduced the speed of the microalgae by 80% in 60 min (from 59 ± 6 $\upmu$m/s at 0 min to 12 ± 4 $\upmu$m/s at 60 min). Similarly, a reduction of 64% in 60 min was observed for the microalgae with 1.77 $\upmu$M Ag^+^ ions (from 61 ± 6 $\upmu$m/s at 0 min to 22 ± 6 $\upmu$m/s at 60 min). These reductions were significantly higher (p-value = 0) than that for the control without Ag^+^ ions (by 21%, from 61 ± 4 $\upmu$m/s at 0 min to 48 ± 4 $\upmu$m/s at 60 min). In addition, we examined the distributions of the instantaneous speeds of the microalgae and observed that Ag^+^ ions resulted in dramatic shifts in the speed distributions. As shown in Fig. 7B, the distributions of the instantaneous speeds appeared as single peaks for the microalgae in the control without being exposed to Ag^+^ ions, while the peaks were centered at 45 $\upmu$m/s. During the observation of 60 min, the distribution remained as a peak, although a shift to slightly lower values ($\sim 29$ $\upmu$m/s) was present, presumably due to small changes in the lighting and temperature conditions. In contrast, the peaks around 45 $\upmu$m/s disappeared for the microalgae exposed to Ag^+^ ions at 1.77 $\upmu$M or 2.35 $\upmu$M (Fig. 7B) as soon as 15 min. Instead, the distributions of the instantaneous speeds of the treated microalgae were maximized at 0 $\upmu$m/s. As the exposure time was longer, the maximum at 0 $\upmu$m/s became increasingly dominant (Fig. 7B).

### Ag^+^ ions increased directional change in microalgae in response to silver ions

**Fig. 8:**
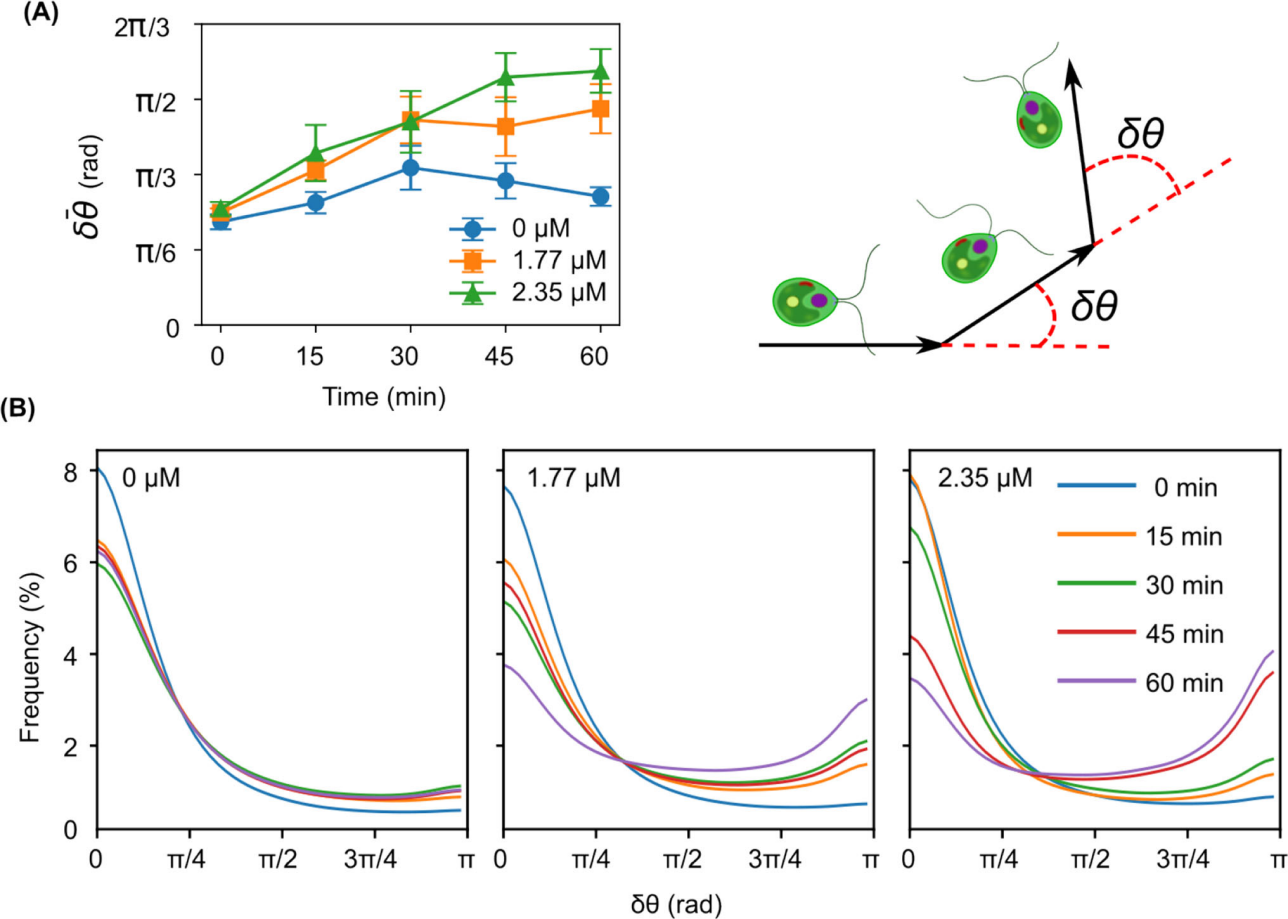
Comparison of the directional changes of the microalgae upon exposure to Ag^+^ ions at different concentrations for different durations. **A** Dependence of the average directional change ($\bar{\delta \theta }$) of the microalgae on the exposure time in the absence (0 $\upmu$M) and presence (1.77 and 2.35 $\upmu$M) of Ag^+^ ions. The sketch on the side illustrates the definition of the directional changes. **B** Distributions of the directional changes of the microalgae at different concentrations of Ag^+^ ions for different durations

We also estimated the directional changes, $\delta \theta$, of the microalgae per step (or per frame of the acquired videos) from their trajectories in the presence of Ag^+^ ions for different durations. As shown in Fig. 8A, we observed that the average directional change increased dramatically by 122% when the microalgae were exposed to 2.35 $\upmu$M Ag^+^ ions for 60 min (from 0.81 ± 0.05 rad/frame at 0 min to 1.8 ± 0.2 rad/frame at 60 min). Similarly, an increase of 92% in 60 min was observed for the microalgae with 1.77 $\upmu$M Ag^+^ ions (from 0.78 ± 0.03 rad/frame at 0 min to 1.5 ± 0.2 rad/frame at 60 min). These increases were significantly higher (p-value $\le$ 10^-173^) than that for the control without Ag^+^ ions (by 24%, from 0.72 ± 0.05 rad/frame at 0 min to 0.89 ± 0.06 rad/frame at 60 min). Examining the distributions of the directional changes of the microalgae showed that Ag^+^ ions resulted in higher frequencies of large directional changes ($\ge \pi /2$ rad/frame or 90$^\circ$/frame). The distributions of the directional changes of the microalgae without being exposed to Ag^+^ ions peaked at 0 rad/frame during the whole observation time (up to 60 min). In contrast, those of the microalgae exposed to 2.35 $\upmu$M Ag^+^ ions for 45 and 60 min peaked at both 0 rad/frame and $\pi$ rad/frame as shown in Fig. 8B. We also note that Ag^+^ ions did not change the symmetry of left/right turns of the microalgae. As shown in Supplementary Figure 2, the signed directional changes showed zero averages and symmetric distributions.

## Conclusions

To summarize, we exposed *C. reinhardtii* microalgae to Ag$^+$ ions of different concentrations and evaluated their effects on the growth, chlorophyll content, and more importantly, the individual motility of the microalgae. We observed that $Ag^+$ ions at concentrations from 0.29 to 0.88 $\upmu$M elongated the lag time of the microalgae, while microalgae exposed to 1.18 $\upmu$M Ag^+^ ions did not grow in 188 hr. On the other hand, we found that, at low concentrations of Ag^+^ ions (0.29 and 0.59 $\upmu$M), the microalgae reached higher densities than those without Ag^+^ ions, showing hormetic responses. Additionally, we measured that Ag^+^ ions at sublethal concentrations increased chlorophyll content in microalgae. More importantly, we measured the swimming motility of individual microalgae under a microscope, tracked their motion based on deep learning algorithms, quantified their speeds and directional changes, and evaluated the effects of Ag^+^ ions on the motility of microalgae. We found that Ag^+^ ions at $\ge$ 1.77 $\upmu$M reduced the speed and increased the directional changes of microalgae in less than 60 min, suggesting slower swimming and larger angular dispersion.

YOLO models have been previously employed for the detection, identification, and classification of microalgae [52, 54, 55, 58]. It was used in this study for tracking the motion and investigating the swimming motility of individual microalgae. Our model demonstrated high accuracy and sensitivity in detecting and tracking individual microalgal cells, allowing us to reliably assess the effects of Ag^+^ ions on microalgal motility. Additionally, we explored the use of Mask R-CNN, another deep neural network architecture for object detection and segmentation [59, 60], which achieved comparable accuracy and sensitivity. Compared to image segmentation based algorithms that we and other researchers used [17, 30, 31, 61–63], one advantage of the deep learning algorithms is that the same trained model can identify and locate microorganisms that are slightly out of focus (in addition to those in focus) without adjusting parameters. We expect that deep learning algorithms have great potential and applications in advancing the studies of freshwater and marine microorganisms and the effects of various environmental perturbations on these microorganisms at the single-cell level. However, custom-trained deep learning models under specific experimental conditions (e.g., particular microscopic settings and background textures) may not generalize effectively to other conditions. Therefore, establishing a collaborative consortium for sharing the standardized deep learning models within the microalgae research community would be highly beneficial.

We observed that Ag^+^ ions significantly hinder the motility of microalgae (Figs. 7 and 8) by reducing the speed and increasing the directional changes. It would be interesting to understand the mechanism of these effects on the microalgal motility. Ag^+^ ions are known to damage microbes multimodally, such as generating ROS, damaging various cellular components (proteins, DNA, and membrane), and interfering with ATP production [11, 35, 64]. It is possible that these damages caused by Ag^+^ ions ultimately affect the motility of the microalgae. On the other hand, a previous study of the effect of Ag^+^ ions on the bacterial motility showed that Ag^+^ ions directly interact with flagellar motors of the bacteria and stall the rotation of the flagellar motors [65]. Thus, another possibility is that microalgal flagellar motors are affected by Ag^+^ ions directly in a similar way. Considering the short time scale of the motility study in this work (1 hr) and relative long doubling time of the microalgae ($\sim$26 hr) [66], it is more likely that Ag^+^ ions directly interfere with the function of the flagellar motor of the microalgae. It would be interesting to investigate the effects of Ag^+^ ions on the beating and dynamics of the microalgal flagella in the future to understand the underlying mechanism.

In this work, 15 min was chosen as the time interval for examining the effects of Ag^+^ ions on the motility of microalgae. It is adequate for the Ag^+^ ions to diffuse around the observation window (the field of view is $\sim$ 200 $\upmu$m, while the size of front lens of the objective is $\sim$ 1 mm). It was also long enough so that residual flows introduced by adding silver nitrate solutions had minimal effects on the microalgae. To show this, we took videos of moving microalgae before, during, and after adding silver nitrate solution in the same video, and examined the distributions of moving directions of the microalgae. As shown in Supplementary Figure 3, the distribution of moving directions of the microalgae was roughly homogeneous in all directions before the addition of 80 $\upmu$L solution. In contrast, clear flow/drift of the microalgae was observed during addition of the solution, and the distribution of the moving directions of the microalgae showed a preference toward one direction. However, the distribution of their moving directions returned to homogeneity after $\sim$320 s (or 5 min).

Trajectories of microalgae were used directly, without smoothing, to calculate their speeds and directional changes and to analyze how their motility responds to Ag^+^ ions. As smoothing has been commonly used to reduce noise in biological motility trajectories [67, 68], we attempted to smooth the trajectories before calculating speeds and directional changes using spline fitting with different regularization parameters ($\lambda$ = 0.1, 1, 10) [69, 70]. We found that our results on the effects of Ag^+^ ions on microalgae motility were robust as the same trends were observed from unsmoothed data and smoothed data with different parameters (Supplementary Figure 4).

It is clear that Ag^+^ ions could completely inhibit the growth of microalgae at high enough concentrations ($\ge$ 1.18 $\upmu$M, Fig. 3A). They can also significantly reduce the motility of the microalgae (Figs. 7 and 8), which is a key behavioral characteristic of microalgae and plays critical roles in their survival and growth. These findings contribute to the mechanistic understanding of the response of living systems to non-equilibrium perturbations by demonstrating the disruption of the mechanical and dynamic behavior of microalgae due to external ionic stressors. Our results also emphasize the necessity of quantifying such interactions from a biophysical point of view for future regulatory approaches of silver compound release into the environment.

Although silver is not an essential mineral, we observed hormetic responses of the microalgae to Ag^+^ ions. Higher densities of microalgae were achieved by exposing them to low concentrations of Ag^+^ ions (Fig. 3A); enhanced levels of chlorophyll content were also measured in microalgae in the presence of Ag^+^ ions (Fig. 4A and B). Others have reported that AgNPs enhanced the growth of sugarcane by increasing the uptaking of macronutrients and micronutrients, which are associated with the biosynthesis and chlorophyll. It would be interesting to quantify how the levels of micronutrients and macronutrients are affected in microalgae by Ag^+^ ions. Additionally, as microalgae have been extensively exploited in various fields, including biofuel production, animal feed, and cosmetics [71–73], it would be exciting to explore the applications of hormetic responses of microalgae to Ag^+^ ions in such fields.

In this study, the concentrations of Ag^+^ ions were higher in the motility assay (1.77 and 2.35 $\upmu$M) than those used in the growth curve assay ($\le 1.18$ $\upmu$M). Little changes were observed within one hour when the microalgae were exposed to Ag^+^ ions at or below 1.18 $\upmu$M. Considering that Ag^+^ ions at 1.18 $\upmu$M completely suppressed the growth of microalgae for at least 188 h, we expect that Ag^+^ at this concentration will significantly impact the motility of microalgae. It would be interesting to investigate how microalgae motility is affected by Ag^+^ ions in longer time scales. On the other hand, we point out that, as the motility of microalgae depends on various factors—including day–night cycles and time points during the day–night patterns [17], stringent controls are necessary to isolate the effects of Ag^+^ ions on their motility. We expect that development of automated devices for both growth monitoring [74, 75] and microscopic imaging [76] will facilitate such studies.

## Supplementary Information

Below is the link to the electronic supplementary material.

Supplementary file 1 (pdf 483 KB)

## Data Availability

The data that support this article are available at https://github.com/ywangua/epje2025.
