# Supplementary material for: Concentration-dependent responses of C. reinhardtii to silver ions: hormetic response in growth and reduction of motility
Source: Eur Phys J E Soft Matter. 2025 Sep 15;48(8-9):56. doi: 10.1140/epje/s10189-025-00521-3 (PMC12436520; doi:10.1140/epje/s10189-025-00521-3)
Supplement: Supplementary file 1 — (pdf 483 KB) [file 10189_2025_521_MOESM1_ESM.pdf]

Supplementary Materials

Concentration-dependent responses of *C. reinhardtii* to silver ions: hormetic response in growth and reduction of motility

Hemanta Pradhan[1], Arpan Poudel[2], Diksha Shrestha[1,3], Ariel Rogers[1], Michael Stewart[1], Amani Jereb[1,3], Jack Harper[1], Ming Li[1], Wen Zhang[3,6], Jingyi Chen[4,5], Yong Wang[1,3,5 *]

[1] Department of Physics, University of Arkansas, Fayetteville, Arkansas, 72701, USA.
[2] Department of Electrical Engineering and Computer Science, University of Arkansas, Fayetteville, Arkansas, 72701, USA.
[3] Cell and Molecular Biology Program, University of Arkansas, Fayetteville, Arkansas, 72701, USA.
[4] Department of Chemistry and Biochemistry, University of Arkansas, Fayetteville, Arkansas, 72701, USA.
[5] Materials Science and Engineering Program, University of Arkansas, Fayetteville, Arkansas, 72701, USA.
[6] Department of Civil Engineering, University of Arkansas, Fayetteville, Arkansas, 72701, USA.

[*] Corresponding author. Email: yongwang@uark.edu.

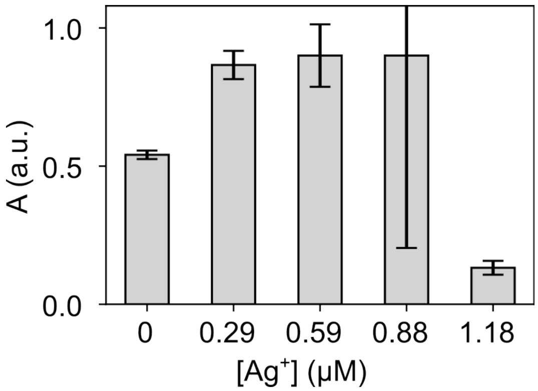

Supplementary Figure 1. Dependence of the fitted asymptotic value / plateau value (A) from the growth curves on the concentration of Ag^+ ions.

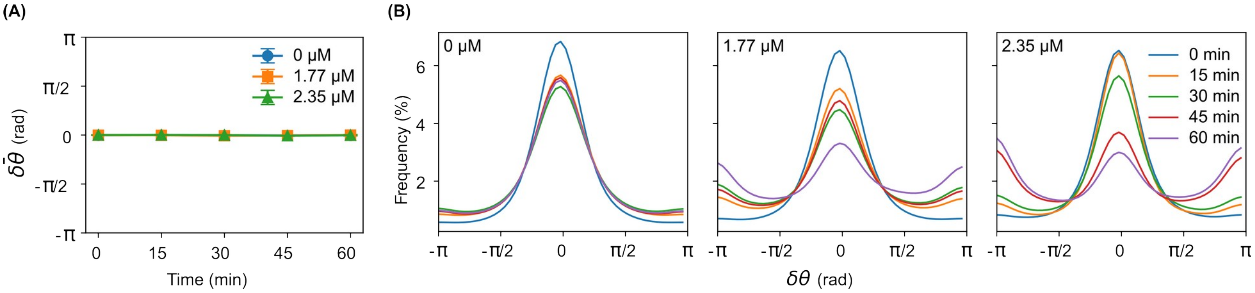

Supplementary Figure 2. Comparison of the signed directional changes of the microalgae upon exposure to Ag^+ ions at different concentrations for different durations. (**A**) Dependence of the average signed directional change of the microalgae on the exposure time in the absence (0 μM) and presence (1.77 and 2.35 μM) of Ag^+ ions. (**B**) Distributions of the signed directional changes of the microalgae at different concentrations of Ag^+ ions for different durations.

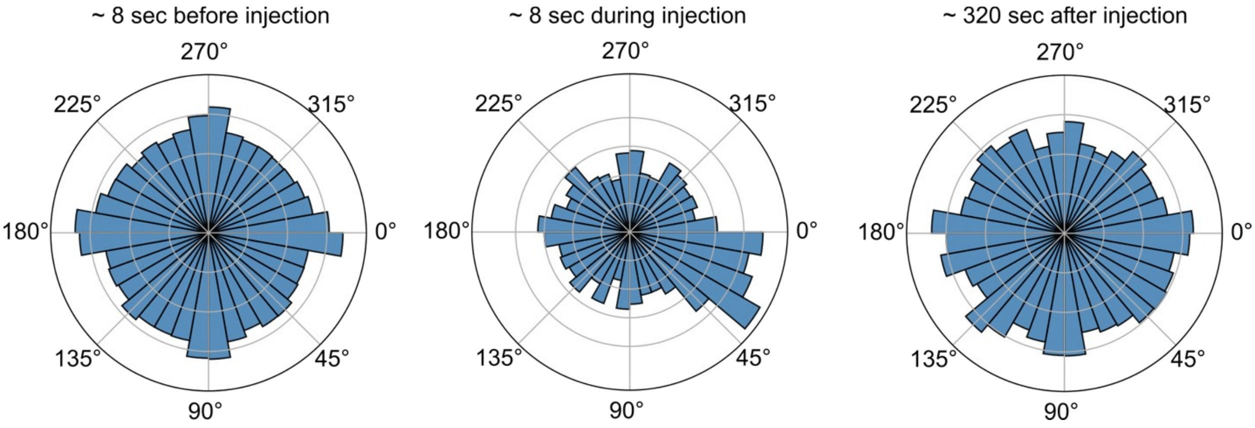

Supplementary Figure 3. Distribution of moving directions of microalgae before, during, and after addition of silver nitrate solution.

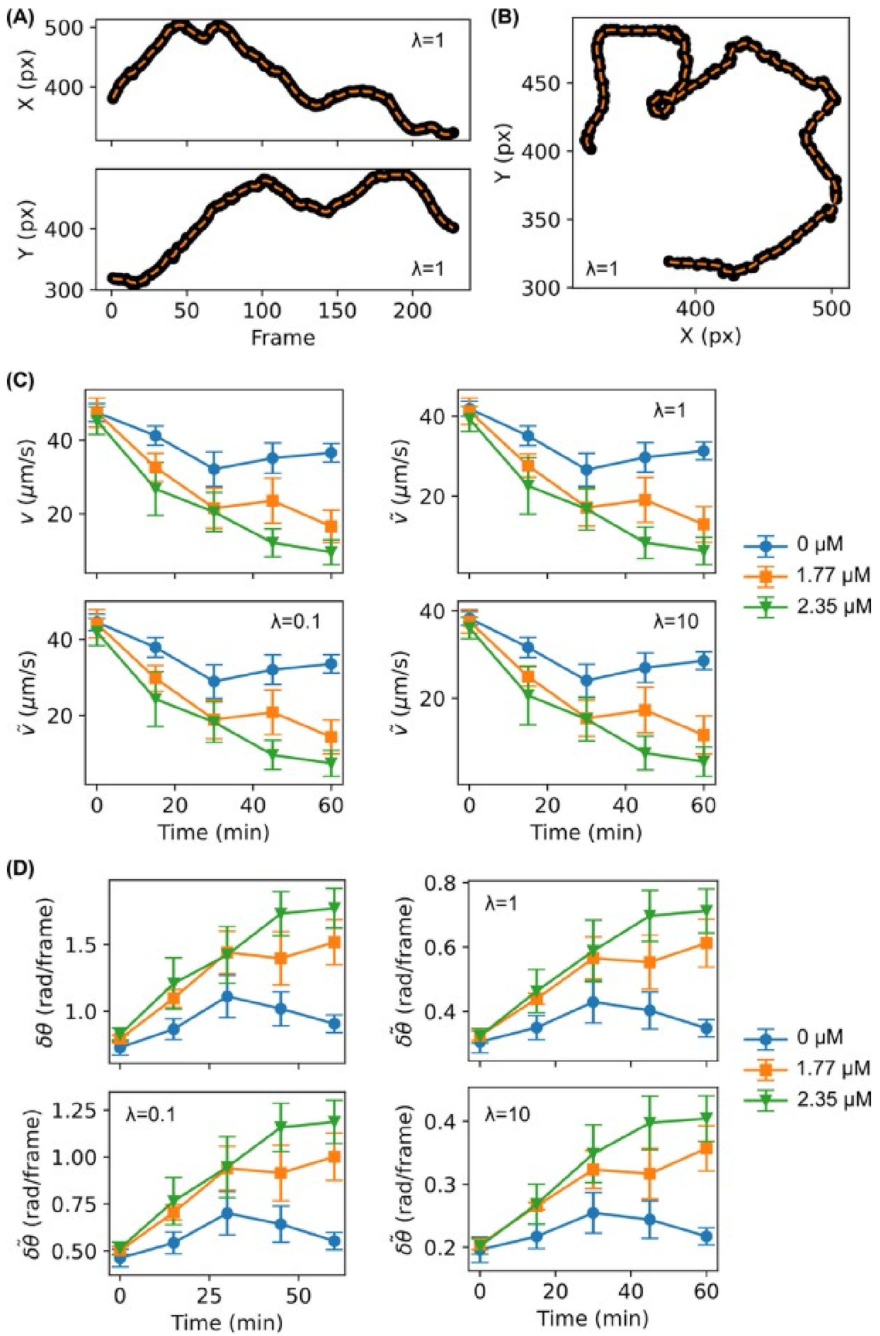

Supplementary Figure 4. Effects of trajectory smoothing based on spline fitting. (**A, B**) An example of a microalgae trajectory before (block dots) and after (orange dashed lines) smoothing based on spline fitting (with regularization parameter λ = 1). (**C**) Dependence of microalgae speeds on exposure duration at different concentrations of Ag^{+} ions for unsmoothed data (v) and smoothed data ($\tilde{v}$) with different regularization parameters. (D) Dependence of microalgae directional changes on exposure duration at different concentrations of Ag^{+} ions for unsmoothed data ($\delta\theta$) and smoothed data ($\widetilde{\delta\theta}$) with different regularization parameters.
